# Health and subjective cognitive decline among Nigerians living with HIV: does perseverance moderate this relationship?

**DOI:** 10.1002/alz70860_107366

**Published:** 2025-12-23

**Authors:** Candidus Nwakasi, Anthony Q Briggs, Darlingtina Esiaka

**Affiliations:** ^1^ University of Connecticut, Storrs, CT, USA; ^2^ NYU Langone Health Center for Cognitive Neurology, New York, NY, USA; ^3^ NYU Alzheimer's Disease Research Center, New York, NY, USA; ^4^ NYU Grossman School of Medicine, New York, NY, USA; ^5^ University of Kentucky College of Medicine, Lexington, KY, USA; ^6^ University of Kentucky Sanders‐Brown Center on Aging, Lexington, KY, USA

## Abstract

**Background:**

Persistent subjective cognitive decline (SCD) exposure is associated with increased risk for Alzheimer' disease and related dementias (ADRD), with this effect exacerbated among people living with HIV. However, there are few studies on SCD among aging Nigerians living with HIV, even as many of them continue to live longer into older age due to advancements in antiretroviral therapy. This study investigated the effects of health status and grit ( a non‐ cognitive trait) on SCD in adult Nigerians with HIV, as previous research suggested that these factors may predict cognitive function.

**Method:**

This cross‐sectional study examined factors influencing mental health care utilization among adult Nigerians living with HIV. Participants (*N* = 117, *M_age_* = 56.06) completed a questionnaire assessing sociocultural factors, mental health seeking, health‐ related measures, non‐ cognitive traits (e.g.grit, resilience), sociodemographic characteristics, and SCD (24‐ item scale; higher score means higher SCD). A multivariate liner regression model was fitted to determine if health status was associated with SCD and the moderation effect of grit on the association. Age, gender, martial status, education and collectivist cultural values were controlled for in the model.

**Result:**

Analysis revealed that better subjective health was negatively associated with SCD (ß =‐ 12.664, p. < . 001), while grit was positively associated with SCD (ß = 1.135, *p* < .001). Those who are married or living together are less likely to report SCD compared to those who are not (ß = 2.907, *p* < .006), and an increased collectivist view (strong sense of social ties) is associated with higher likelihood of SCD (ß = 2.907, *p* < . 008). Moderation analysis also showed that grit could reduce the effect of subjective health on SCD (ß = ‐0.405, *p* < .001).

**Conclusion:**

The findings may be used as a groundwork for future research examining the role of health and non‐ cognitive traits and cognitive decline in older adults living with HIV. It can also inform cognitive health intervention programs for middle ‐aged and older adults with HIV.

